# Identification and analysis of major flavor compounds in radish taproots by widely targeted metabolomics

**DOI:** 10.3389/fnut.2022.889407

**Published:** 2022-07-18

**Authors:** Shiyong Mei, Zhengjin He, Jifang Zhang

**Affiliations:** ^1^Institute of Bast Fiber Crops, Chinese Academy of Agricultural Science, Changsha, China; ^2^Center for Southern Economic Crops, Chinese Academy of Agricultural Science, Changsha, China

**Keywords:** *Raphanus sativus*, metabolite profiling, taste, taproot, landraces

## Abstract

Radish (*Raphanus sativus* L.) is an important Brassicaceous vegetable crop that is cultivated worldwide. The taste of radish can be described as pungent, sweet, and crisp. At present, the metabolic characteristics leading to differences in radish taste remain unclear, due to the lack of large-scale detection and identification of radish metabolites. In this study, UPLC-MS/MS-based targeted metabolome analysis was performed on the taproots of eight radish landraces. We identified a total of 938 metabolites, and each landrace exhibited a specific metabolic profile, making it unique in flavor and quality. Our results show that taste differences among the taproots of different radish landraces can be explained by changes in composition and abundance of glucosinolates, polyphenols, carbohydrates, organic acids, amino acids, vitamins, and lipids. This study reveals the potential metabolic causes of variation in the taste and flavor of radish taproots.

## Introduction

Radish (*Raphanus sativus* L., 2n = 2x = 18) belongs to the *Brassicaceae* family and *Raphanus* genus. It is the most widely cultivated and consumed vegetable in East Asia (China, Japan, and Korea), as well as an important economic crop worldwide. Radish cultivation China has a history of more than 2000 years (1). The long-term evolution, domestication, and artificial selection led to hundreds of distinctive landraces, with variation in root sizes, shapes, colors, and flavors (2). Radish can be used for various purposes. In northern China, some green-skinned radish cultivars, with sweet and crisp flavors, are consumed as fruits (3). In southern China, the red-skinned types, with a pungent taste, are mainly used for food coloring, cosmetics, and the medical industry (4–6). The mild white-skinned types are mainly used for cooking and stewing. Some varieties with low water content and high compact texture can be used for cooking and food processing (3).

Radishes are rich in bioactive metabolites that influence its taste. In previous studies, important secondary metabolites, such as glucosinolates (GS) and flavonoids, have attracted much attention and are characteristic in some unique Chinese radish cultivars (7–11). Different cultivars and growing conditions resulted in high variation in the profiles and content of these metabolites (8, 11, 12). In addition, fruit and vegetable flavor was greatly affected by the concentrations of carbohydrates, organic acids, and polyphenols (13–15). Studies have shown the compositions and abundance of amino acids also affected the taste (16–18). However, little information is available on the profiles of carbohydrates, amino acids, and organic acids in different radish landraces.

To date, there are few large-scale detection, identification, and quantification methods for the flavor substances in radish taproots. The reported methods mainly focus on high-performance liquid chromatography with diode-array detection (HPLC+DAD), HPLC alone or liquid chromatography-electrospray ionization-mass spectrometry (LC-ESI-MS) (19). Targeted metabolomics analysis, based on ultra-performance liquid chromatography-tandem mass spectrometry (UPLC-MS/MS), is a fast and reliable method for large-scale and comparative metabolomics studies (20).

In this study, we aimed to identify a wide range of metabolites that could contribute to taste variations among radish landraces. The UPLC-MS/MS method was performed to identify and quantify metabolites of eight radish cultivars. These metabolites included glucosinolates, phenolic acids, organic acids, lipids, vitamins, and amino acids et al. Our results provide useful data for clarifying the taste differences among radish cultivars and could further guide breeding research.

## Materials and methods

### Plant materials and chemicals

**Figure 1:**
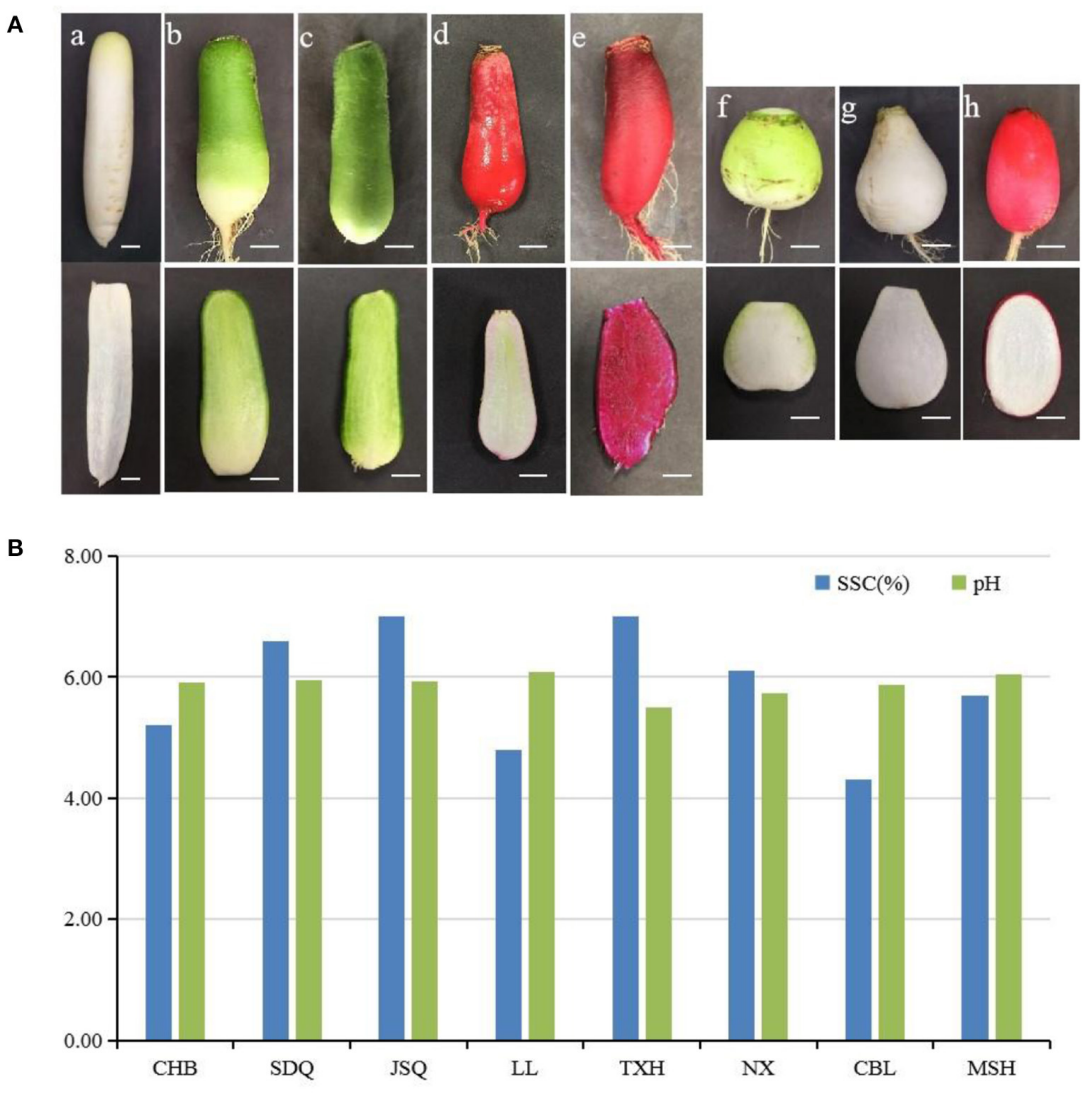
**(A)** Root type characteristics of eight radish landraces. **(a)**, Chunchangbai (CHB); **(b)**, Shandongqing (SDQ); **(c)**, Jiangsuqing (JSQ); **(d)**, Xuzhoulaluobo (LL); **(e)**, Touxinhong (TXH); **(f)**, Nanxiang (NX); **(g)**, Chunbulao (CBL); **(h)**, Manshenhong (MSH). Scale bar = 2 cm. **(B)** Soluble solids content (SSC) and acidity (pH) of eight radish landraces.

One double haploid (DH) line and seven advanced inbred lines of radish landraces were used in this study: Chunchangbai (CHB, DH) from Korea and Chunbulao (CBL) from Guangdong (China) with white skin and fleshed taproot, Shandongqing (SDQ) from Shandong and Jiangsuqing (JSQ) from Jiangsu (China) with green taproot flesh and green skin, Touxinhong (TXH) from Sichuan (China) with dark red skin and fleshed taproot, Nanxiang (NX) from Hubei (China) with green taproot skin and white flesh, Manshenhong (MSH) from Sichan (China) and Xuzhoulaluobo (LL) from Jiangsu (China) with light red taproot skin. CHB has a long cylindrical taproot. SDQ, JSQ, LL and TXH have an average-sized cylindrical taproot. NX, MSH and CBL have short cylindrical or oval taproots (Figure 1). All plants were grown in pots at the Institute of Best Fiber Crops at the Chinese Academy of Agricultural Sciences in December of 2020. Developed taproots (80 d after sowing) were harvested, washed with running tap water, and then cleaned with sterile water. The taproots were cut into pieces and stored at −80°C until further analysis. Each sample consisted of three replicates, and each replicate contained four individual plants.

Gradient grades of methanol, acetonitrile, and formic acid were purchased from Merck Company, Germany (www.merck-chemicals.com). The internal standard L-2-chlorophenylalanine was bought from J&K Scientific Co., Ltd (www.jkchemical.com/).

### Sample preparation and extraction

The sliced radish taproots were freeze-dried in a vacuum freeze-dryer (Scientz-100F, Ningbo, China) and then ground with zirconium beads at 30 Hz for 1.5 min (MM 400, Retsch, Haan, Germany). Then, 100 mg homogenized, sieved and lyophilized powder was weighed and dissolved with 1.2 mL 70% methanol extract. The sample was vortex for 30 s every 30 min, six times in total. The mixture was extracted overnight at 4°C. After centrifugation (12,000 rpm, 10 min), the supernatant was absorbed and filtered by SCAA-104 (ANPEL, Shanghai, China) with a 0.22-μm pore size. Then, the supernatant was qualitatified by high resolution mass spectrometry AB SCIEX 6600 QTOF, and AB SciEX 4500 Q TRAP UPLC/MS/MS system.

The fresh juice of each landrace was extracted for soluble solids content measurement using a Brix refractometer (LC-DR-53B, Lichen Co., Ltd., Shanghai, China); pH measurements were recorded using a pH meter (PHS-3C, INESA Scientific Instrument Co., Ltd., Shanghai, China).

### UPLC conditions

UPLC was performed on an Agilent SB-C18 column (1.8 μm, 2.1 mm^*^100 mm). The mobile phase consisted of solvent A (pure water with 0.1% formic acid) and solvent B (acetonitrile with 0.1% formic acid). The samples were separated *via* a solvent gradient program, starting at 95% A and 5% B, followed by a linear gradient of 5% A and 95% B over 9 min, and maintained the final composition for 1 min. A composition of 95% A, 5.0% B was adjusted within 1.10 min and kept for 2.9 min. The flow velocity was set as 0.35 ml per min. The column oven was set to 40°C. The injection volume was 4 μl. Subsequently, the effluent was connected to an ESI-triple Quadrupole Linear Ion Trap (QTRAP)-MS.

### ESI-Q TRAP-MS/MS

We used an AB4500 Q TRAP UPLC/MS/MS system, equipped with linear ion trap (LIT) and triple quadrupole (QQQ) for metabolite detection. An ESI Turbo Ion-Spray interface was operated in positive and negative ion modes and controlled by Analyst 1.6.3 software (AB Scitex, Foster City, CA, USA). The ESI source operation parameters were as follows: ion source (turbo spray, 550°C, 5,500 V /−4,500 V); gas I, gas II, and curtain gas were set at 50, 60, and 25.0 psi, respectively. The collision-activated dissociation was set to “high.” For QQQ and LIT modes, 10 μmol L^−1^ and 100 μmol L^−1^ polypropylene glycol solutions were used for instrument tuning and mass calibration, respectively. QQQ scans were acquired in the MRM experiments. The collision gas (nitrogen) was set to “medium.” For each MRM transition, de-clustering potential (DP) and collision energy (CE) were further optimized. A specific set of MRM transitions were monitored, according to the eluted metabolites at each period.

### MS data and statistical analysis

MS data acquisition and processing were conducted based on previous methods (21). Metabolites were annotated based on the self-built database MWDB (Wuhan Metware Biotechnology Co., Ltd., Wuhan, China). Metabolite identification was based on the accurate mass of metabolites, MS2 fragments, MS2 fragments isotope distribution and retention time (RT). Through the company's self-built intelligent secondary spectrum matching method, the secondary spectrum and RT of metabolites in our samples were matched intelligently with the secondary spectrum and RT of the company's database one by one. The MS tolerance and MS2 tolerance were set to 2 ppm and 5 ppm, respectively. Metabolites that did not have standard products were compared with MS2 spectra in public databases or literature. Some of the metabolites without standard secondary spectra were inferred based on experience (22).

The metabolic data were processed using multivariate statistical analysis methods, including unsupervised principal component analysis (PCA), supervised multiple regression orthogonal partial least squares discriminant analysis (OPLS-DA), and hierarchical clustering analysis (HCA). PCA was performed using the statistics function prcomp within R v3.5.1 (www.r-project.org). Logarithmic transformation (log2) was performed on the MS data before OPLS-DA, and the mean was centered. OPLS-DA was carried out using R package MetaboAnalystR. The *p* and fold change values were 0.05 and 2.0, respectively. HCA was carried out by R package pheatmap. The number of differential metabolites was illustrated by Venn diagrams among radish samples. Using the Kyoto Encyclopedia of Genes and Genomes (KEGG) database, with a *p*-value < 0.01, the differential metabolites between TXH and the other radish landraces were studied. All data were plotted by GraphPad Prism v6.01 (GraphPad Software Inc., La Jolla, CA, USA).

## Results

### Morphological and geographical taproot differences

The eight radish landraces were grown under uniform conditions. The morphology of the radish taproots, particularly the shape, color, and sizes, were obviously different (Figure 1A). The soluble solids content (SSC) of radish landraces ranged from 4.3% to 7.0%. CBL had the lowest SSC 4.30%, TXH and JSQ had the highest SSC 7.00% (Figure 1B). The pH of radish landraces ranged from 5.49 to 6.09 (Figure 1B).

### Targeted metabolic profiling

To understand the taste differences among radish landraces, targeted metabolite analysis using UPLC–MS/MS was performed to identity comprehensive metabolic profiles of radish taproots. A total of 938 metabolites were identified, including 156 lipids, 87 phenolic acids, 87 organic acids, 118 amino acids, and derivatives that are likely to contribute to radish taste. Other primary and secondary metabolites were also identified (Supplementary Table S1).

### Multivariate analysis revealed differences among the identified metabolite profiles

**Figure 2:**
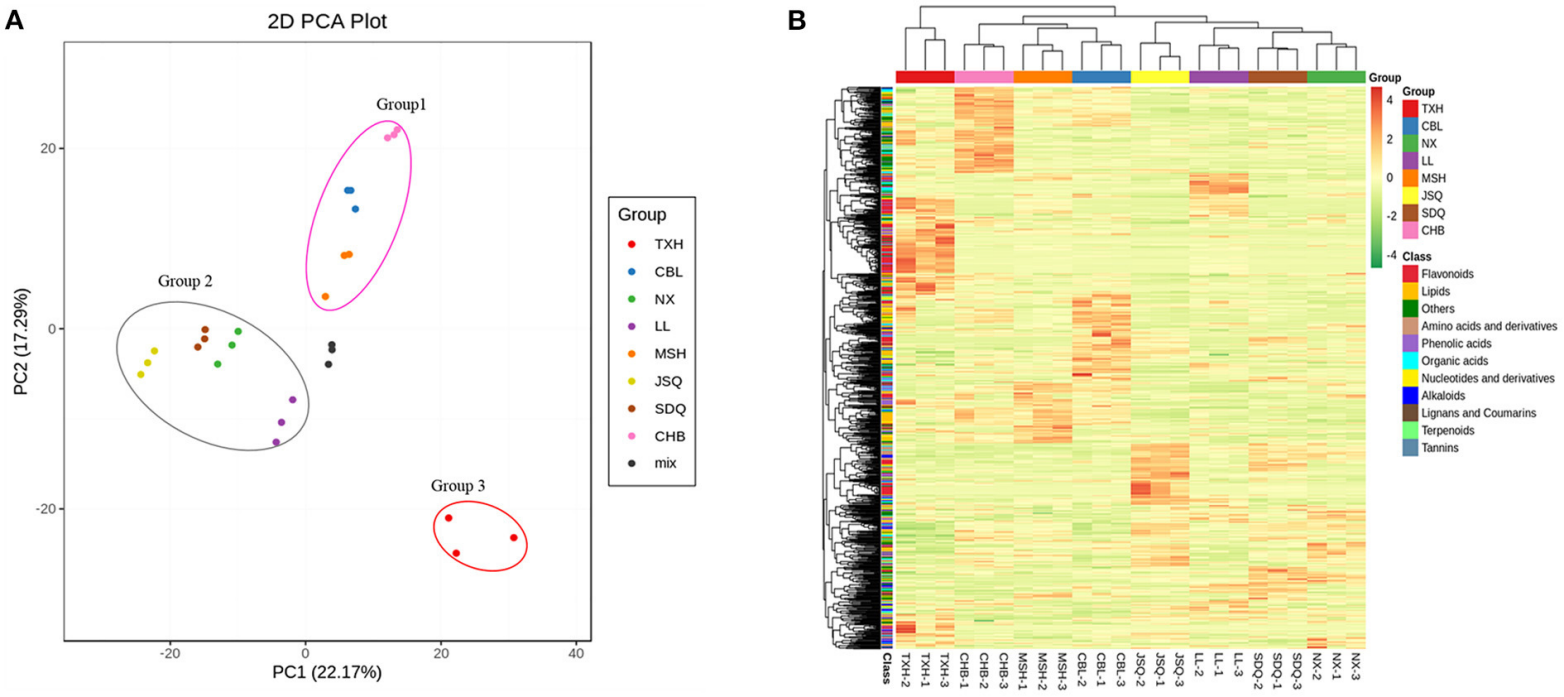
PCA **(A)** and heat map visualization **(B)** of the relative differences in the metabolite profiles among different radish landraces. The sampling groups are color-coded as follows: red, TXH; green, NX; blue, CBL; purple, LL; pink, CHB; orange, MSH; yellow, JSQ; brown, SDQ; mix, the mixture of the radish sample extracts. In the heat map, each sample is represented by a column, and each metabolite is displayed in a row. Progressive colors show the enrichment level of each metabolite from low (green) to high (red).

To assess the differences among the metabolite profiles of the eight radish landraces, we performed multivariate statistics. PCA was performed to clarify the internal structure of multiple variables on the 938 metabolites. The mixture of radish sample extracts was used as a quality control (QC) sample. The QC samples gathered in the same area, suggesting that they had similar metabolic profiles, and the analysis was stable and repeatable. The result showed the eight samples were divided into three different groups, indicating that each group had a relatively different metabolic profile. Group 1 included CHB, CBL, and MSH with white-fleshed taproot. Group 2 included JSQ, SDQ, NX, and LL, with green and white-fleshed taproot. Group 3 consisted of TXH with red skin and red-fleshed taproot (Figure 2A).

To remove the effects of quantity on pattern recognition, we performed a log10 transformation on the peak areas of each metabolite and conducted HCA (Figure 2B). This analysis directly reflects the differences in metabolites between and within different groups. TXH, in group 3, was rich in flavonoids and carbohydrates metabolites. CHB, in group 1, was rich in tannins, organic acids, lipids, and other metabolites. MSH was rich in phenolic acids, lipids, and tannins. CBL was rich in flavonoids, lipids, nucleotides, and derivatives. LL, in group 2, was rich in organic acids, flavonoids, amino acids, and derivatives. Thus, the results of the PCA and HCA suggest these eight landraces had distinct metabolite profiles.

### Differential metabolite analysis

A pairwise comparison of the eight radish landraces was carried out to determine the metabolites that lead to differences in taste. In OPLS-DA analysis (Supplementary Figures S1A–G), CBL, CHB, JSQ, LL, MSH, NX, and SDQ were clearly separated from TXH, indicating that there are major distinctions in metabolic profiles between different landraces.

**Figure 3:**
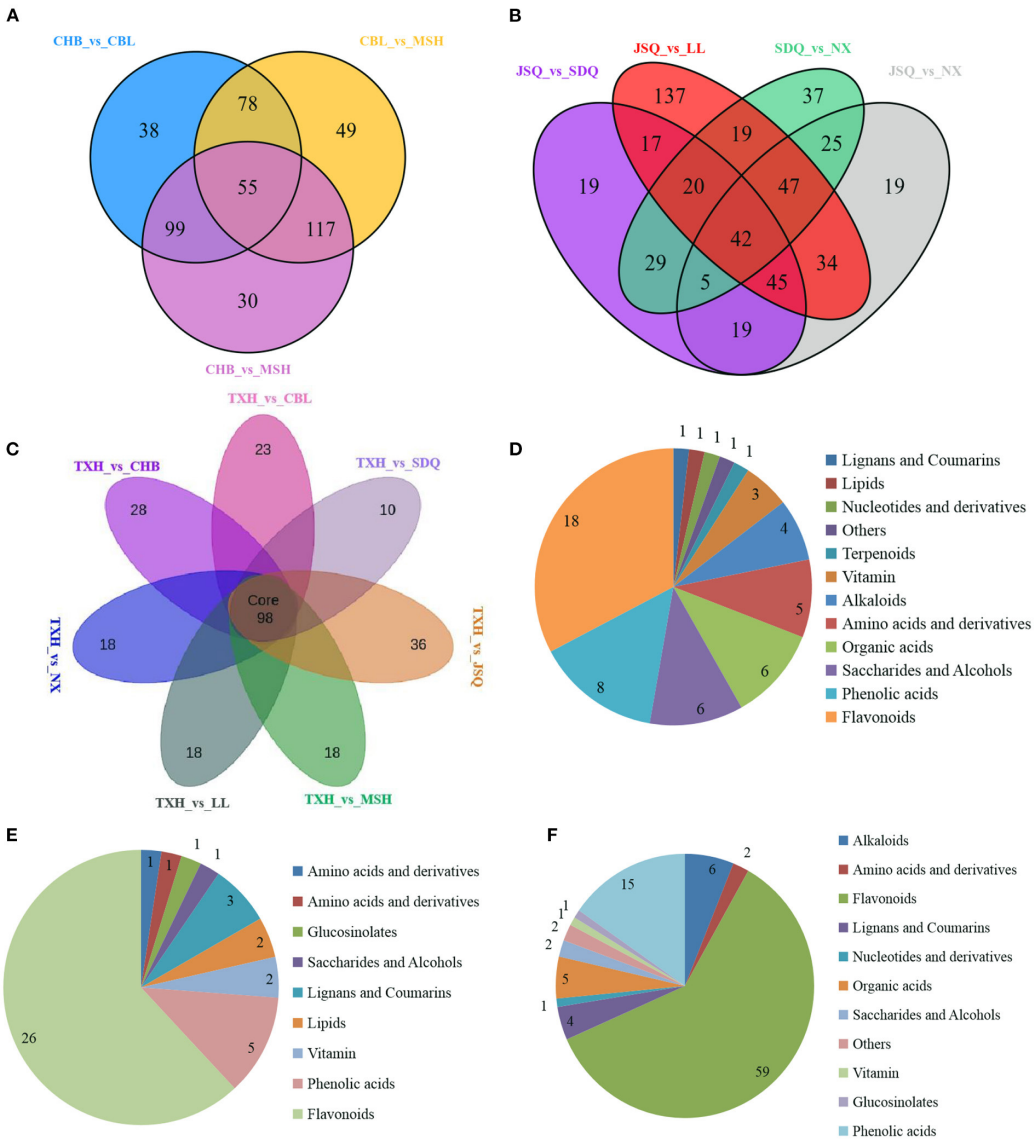
Venn diagrams display the overlapping and landrace-specific differential metabolites of group 1 landraces **(A)**, group 2 landraces **(B)** and all eight landraces **(C)**, respectively. Pie charts **(D–F)** show the biochemical categories of the differential metabolites. **(D)** shows the overlap metabolites of group 1 landraces; **(E)** represents the overlap metabolites of group 2 landraces; **(F)** displays the overlap metabolites of eight landraces.

To identify differential metabolites among the eight landraces, a fold change ≥ 2.0 (higher) or ≤0.5 (lower) was set, as well as variables identified as important in the projection (VIP > 1) scores, to select metabolites of interest. The screening results are presented as volcano plots (Supplementary Figures S2A–G) and Venn diagrams (Figures 3A–F). For landraces in group 1, there were 343 differential metabolites (148 higher and 195 lower) in CBL, compared to TXH; 346 (139 higher and 207 lower) in CHB, compared to TXH; and 328 (118 higher and 210 lower) in MSH, compared to TXH (Table 1). A total of 55 differential metabolites, including 18 flavonoids, 8 phenolic acids, 6 organic acids, and 6 saccharides and alcohols differed between the three landraces (Figures 3A,D; Supplementary Table S2).

**Table 1:** Differential metabolites among the eight landraces.

**Metabolite class**		**TXH_vs_CBL**	**TXH_vs_CHB**	**TXH_vs_JSQ**	**TXH_vs_LL**	**TXH_vs_MSH**	**TXH_vs_NX**	**TXH_vs_SDQ**
Alkaloids	up	13	17	17	12	13	13	14
	down	2	8	4	5	6	5	8
Amino acids and derivatives	up	17	24	42	22	18	39	36
	down	8	13	6	5	20	3	8
Flavonoids	up	31	11	46	11	15	13	42
	down	90	95	99	93	97	104	93
Lignans and coumarins	up	12	4	5	3	12	6	5
	down	5	12	5	12	7	9	5
Lipids	up	14	17	17	7	16	7	14
	down	8	7	30	9	10	14	15
Nucleotides and derivatives	up	11	6	8	3	4	1	2
	down	8	10	13	10	11	18	13
Organic acids	up	11	21	16	22	11	13	16
	down	12	11	17	16	10	11	13
Phenolic acids	up	24	12	12	10	18	14	16
	down	28	37	33	41	27	39	39
Tannins	up	0	0	0	0	0	0	0
	down	1	1	1	1	0	0	1
Terpenoids	up	2	0	5	1	1	3	3
	down	1	3	0	0	3	2	0
Others	up	2	1	1	0	2	1	1
	down	2	2	3	2	4	3	4
Saccharides and alcohols	up	3	10	6	10	4	2	4
	down	23	2	20	15	12	17	21
Glucosinolates	up	6	11	0	4	3	9	11
	down	6	1	7	12	1	6	3
Vitamin	up	2	5	4	2	1	7	5
	down	1	5	4	1	2	3	2
Total	up	148	139	179	107	118	128	169
	down	195	207	242	222	210	234	225
	sum	343	346	421	329	328	362	394

With respect to the landraces involved in group 2, there were 421 differential metabolites (179 higher and 242 lower) in JSQ, compared to TXH; 329 (107 higher and 222 lower) in LL, compared to TXH; 362 (128 higher and 234 lower) in NX, compared to TXH; and 394 (169 higher and 225 lower) in SDQ, compared to TXH (Table 1). A total of 42 differential metabolites, including 26 flavonoids and 5 phenolic acids differed between the four landraces (Figures 3B,E; Supplementary Table S3). Furthermore, a total of 98 different metabolites were found in all eight landraces, including 59 flavonoids, 15 phenolic acids, and 6 alkaloids (Figures 3C,F; Supplementary Table S4).

The 938 differential metabolites among eight landraces were mapped to the KEGG database to obtain detailed information about the metabolic pathways (Supplementary Figures S3A-G). The enrichment analysis of identified metabolites that differ from TXH were mainly involved in flavonoid biosynthesis, phenylpropanoid biosynthesis, flavone and flavonol biosynthesis, and phenylalanine metabolism (Supplementary Table S5).

### GS, organic acids, carbohydrates, polyphenols, and amino acids

In this study, we mainly focused on the classes of metabolites considered to be the major contributors of the difference in radish taproot taste. Each category of metabolite was analyzed based on the fold changes ≥ 2 or ≤0.5 and VIP values > 1, compared with that of TXH.

#### Glucosinolates

GS are sulfur- and nitrogen-containing metabolites common in the order Brassicales, and their degradation products have unique health benefits, as well as pest deterrent properties (23). The content of GS has a great influence on the unique flavor of *Brassica* vegetables, such as turnip (*B. rapa*) and broccoli (*B. oleacrea*) (24, 25). GS can be classified into aliphatic, aromatic, and indole GS, according to their precursor amino acids (26). GS in the taproots significantly affect the pungent taste and quality of fresh radish (23).

In our study, a total of 38 GS was detected, including 25 aliphatic GS, 8 aromatic GS, and 5 indole GS. Based on fold changes and VIP values, the concentration of 4-phenylbutylglucosinolate was most abundant in JSQ, followed by SDQ, CBL, and LL. It was the lowest in TXH. The concentration of sulforaphane was highest in CBL, MSH, and CHB, followed by LL and SDQ. It was the lowest in TXH, NX, and JSQ. The concentration of 2-hydroxy-2-methylpropylglucosinolate was more abundant in TXH, followed by JSQ, LL, MSH, NX, and SDQ. Five kinds of GS, including1-ethyl-2-hydroxyethyl GS, 5-hexenyl GS, gexyl GS, 4-methylpentyl GS, and 3-methylpentyl GS, were most abundant in LL. Nine kinds of GS, including 3-methylbutyl GS, 3-methylsulfinylpropyl GS, 6-sinapoyl-1-thioglucoside of 4-methylthiobut-3-enyl thioglucoside, 7-(methylsulfinyl) heptyl GS, 6-methylsulfinylhexyl GS, 4-hydroxyindol-3-ylmethyl GS, 3-indolylmethyl GS, 4-hydroxy-3-indolylmethyl GS, and 2-methylbutyl GS, were most abundant in SDQ. Five kinds of GS, including 6-(p-hydroxyphenylacrylic acid)-1-GS of 4-methylsulfinyl-3-butenyl thio-glucoside, 2(R)-hydroxy-2-phenylethyl GS, sinalbin, sinigrin, and 3-phenylpropyl GS, were most abundant in CHB (Supplementary Table S6).

#### Organic acids

We identified 87 organic acids in radish taproots, which could explain the weak acidity (pH5.49–6.04) of radish taproots. Twelve organic acids, including 3-ureidopropionic acid, succinic semialdehyde, abscisic acid, 2-hydroxyisocaproic acid, DL-pipecolic acid, methanesulfonic acid, phosphoenolpyruvate, piperonylic acid, L-citramalic acid, glutaric acid, 6-aminocaproic acid, and jasmonic acid were highly abundant and were more prevalent in CHB, compared with the other samples. Twelve organic acids, including 3-methylmalic acid, 2-acetyl-2-hydroxybutanoic acid, 2-propylsuccinic acid, adipic acid, suberic acid, quinic acid, pyrrole-2-carboxylic acid, 2-methylglutaric acid, 3-hydroxyglutaric acid, methylenesuccinic acid, 2-hydroxyglutaric acid, and pimelic acid, were highly abundant in LL. 2-Hydroxyphenylacetic acid and iminodiacetic acid were most abundant in NX. Six organic acids, including 1-pyrroline-4-hydroxy-2-carboxylic acid, 5-acetamidopentanoic acid, fumaric acid, 1-aminocyclopropane-1-carboxylic acid, 6-aminocaproic acid, and aminomalonic acid, were present in high concentrations, and their concentrations were significantly higher in JSQ. 2-Picolinic acid and isocitric acid were the most abundant in SDQ. Phenylpyruvic acid, phenyllactate, 4-pentenoic acid, and tiglic acid were most abundant in MSH. 2-Hydroxy-3-phenylpropanoic acid, creatine, 2-hydroxyisocaproic acid, and 3-guanidinopropionic acid were most abundant in CBL. Fourteen organic acids, including D-lactic acid, 2-methyl-3-oxosuccinic acid, 2-hydroxyhexadecanoic acid, succinic acid, citric acid, methylmalonic acid, 4-guanidinobutyric acid, 3-hydroxybutyric acid, D-galacturonic acid, malonic acid, (-)-jasmonoyl-L-isoleucine, α-ketoglutaric acid, DL-glyceraldehyde-3-phosphate, and oxalic acid, were most abundant in TXH, compared with the other samples (Supplementary Table S7).

#### Carbohydrates

We identified 72 carbohydrates in radish taproots. Among these carbohydrates, D-(-)-threose, helecin, 1,6-anhydro-β-D-glucose, D-(+)-cellobiose, D(+)-melezitose, O-rhamnoside, dambonitol, D-arabinose, D-fructose, D-galactaric acid, D-galactose, D-glucose, D-mannose, D-saccharic acid, D-sorbitol, erythrose, gluconic acid, isomaltulose, L-xylose, N-acetyl-D-mannosamine, and raffinose were most abundant and enriched in TXH. Eleven carbohydrates, including 3-phospho-D-glyceric acid, D-fructose-1, 6-biphosphate, D-glucoronic acid, D-glucosamine, D-maltose, D-pinitol, D-saccharic acid, glucose-1-phosphate, N-acetyl-D-glucosamine-1-phosphate, and sedoheptulose, were more prevalent in CHB than the other landraces. 2-Dehydro-3-deoxy-L-arabinonate and D-arabinono-1,4-lactone were more abundant in LL than the other samples. 3-Methyl-1-pentanol, D-melezitose, and L-gulono-1, 4-lactone were more abundant in CBL. Turanose, lactobiose, and D-lactulose were more abundant in MSH. D-Panthenol, maltotriose and ribitol were highly abundant and had accumulated in SDQ. Sucrose-6-phosphate was more prevalent in JSQ and NX than the other samples (Supplementary Table S8).

#### Polyphenols

Research shows that polyphenols cause bitterness, color, and astringency in beer and other beverages (15). These compounds can be classified as hydroxycinnamic and hydroxybenzoic acid derivatives (phenolic acids), flavanols, flavanol glycosides, and prenylated flavonoids (27). Studies have shown that several phenolics, including ferulic acid, p-coumaric acid, and protocatechuic acid can cause astringency (28). Flavanol monomers, such as catechin and epicatechin, are shown to be more bitter than astringent (29, 30).

In our study, 2-hydroxycinnamic acid was most abundant in MSH and CBL, followed by CHB and SDQ. The lowest concentration was found in TXH and LL. 4-Hydroxybenzoic acid was significantly reduced in CHB, JSQ, LL, NX, and SDQ. 5-Glucosyloxy-2-hydroxybenzoic acid methyl ester was significantly more abundant in LL. 4-O-Glucosyl-4-hydroxybenzoic acid was significantly reduced in CHB, JSQ, LL, MSH, NX, and SDQ. Dihydroferulic acid, ferulic acid, and isoferulic acid were significantly reduced in LL, NX, and SDQ. p-Coumaric acid methyl ester and p-coumaric acid-4-O-glucoside were more abundant in TXH. p-Coumaric acid was most abundant in MSH, followed by CBL and CHB. The lowest concentration was found in LL. Protocatechuic acid-4-O-glucoside was significantly reduced in MSH and SDQ. Epicatechin was abundant in MSH, CBL, CHB, NX, JSQ, LL, and SDQ but absent in TXH. Catechin gallate was most abundant in MSH, followed by CBL, JSQ, and LL. Epicatechin gallate was significantly enriched in NX (Supplementary Table S9).

#### Amino acids

We identified 118 amino acids in the radish taproots. Among them, 34 were most abundant in JSQ, including 3-cyano-L-alanine, 5-oxo-L-proline, 5-oxoproline, cis-4-hydroxy-D-proline, cyclo (Phe-Glu), cycloleucine, DL-tryptophan, L-allo-isoleucine, L-arginine, L-arginine (hydrochloride), L-aspartic acid, L-azetidine-2-carboxylic acid, L-citrulline, L-cystine, L-glutamine, L-homomethionine, L-isoleucine, L-leucine, L-methionine, L-norleucine, L-ornithine, L-phenylalanine, L-tryptophan, L-valine, L-γ-glutamyl-L-leucine, N-acetyl-DL-phenylalanine, N-acetyl-L-tyrosine, N-alpha-acetyl-L-asparagine, nicotianamine, N-α-acetyl-L-ornithine, trans-4-hydroxy-L-proline, γ-glutamyl-L-valine, γ-glutamylmethionine and γ-glutamylphenylalanine. Seven amino acids, such as 10-formyltetrahydrofuran, 5-aminovaleric acid, L-glutamine-O-glycoside, L-homocitrulline, methiin, N-acetyl-beta-alanine and S-adenosylmethionine were highly abundant and prevalent in CHB. Seven amino acids, including 2, 6-diaminooimelic acid, histamine, L-lysine, L-tyrosine, S-methyl-L-cysteine, γ-glutamyltyrosine, and γ-L-glutamyl-S-methyl-L-cysteine were most abundant in NX. Five amino acids, including 1-methylpiperidine-2-carboxylic acid, cyclo (Ala-Gly), L-isoleucyl-L-aspartate, L-prolyl-L-leucine and N-acetyl-L-aspartic acid were most significantly enriched in CBL. Three amino acids, including L-aspartic acid-O-diglucoside, L-cysteine, and L-proline, were most abundant in SDQ. Three amino acids, including N6-acetyl-L-lysine, N-acetyl-L-phenylalanine, and trimethyllysine, were most significantly enriched in MSH. Nicotinuric acid, N-acetyl-L-threonine, S-(5'-Adenosyl)-L-methionine chloride, and γ-Glu-Cys were most abundant in LL. S-allyl-L-cysteine was most abundant in TXH (Supplementary Table S10).

#### Vitamin

We identified 24 vitamins in radishes. Among these vitamins, thiamine (vitamin B1) was highly abundant in CHB. Dehydroascorbic acid and pyridoxine-5'-O-glucoside were most abundant in JSQ, followed by CHB, NX, and SDQ. 4-Pyridoxic acid-O-glucoside, D-pantothenic acid, calcium pantothenate, and 4-pyridoxic acid were most abundant in NX. Biotin was the most abundant in LL (Supplementary Table S11).

#### Lipids

Lipids provide energy, give special flavor and taste to food, and are essential substances in human health. In this study, we identified 76 lipids. Based on fold changes and VIP values, nine lipids, including lysoPE 15:1 (2n isomer), lysoPC 17:2, lysoPC 18:1 (2n isomer), lysoPC 15:1, lysoPC 14:0, docosanoic acid, lysoPC 18:2 (2n isomer), lysoPC 18:2 and lysoPC 18:1, were most abundant in MSH. Nine lipids, including 2R-hydroxy-9Z, 12Z, 15Z-octadecatrienoic acid, 3S-hydroxy-9Z, 11E, 15Z-octadecatrienoic acid, lysoPE 16:0 (2n isomer), lysoPC 15:0, lysoPC 12:0, lysoPC 16:0, LysoPE 15:0 (2n isomer), lysoPC 15:0 (2n isomer), and 9, 12, 13-TriHOME, were most abundant in CHB. Eight lipids, including 9, 10, 11-trihydroxy-12-octadecenoic acid, 7S, 8S-DiHODE, 13-KODE, 12, 13-DHOME, (9Z, 11E)-octadecadienoic acid, 9-hydroxy-10, 12, 15-octadecatrienoic acid, and 9, 10, 13-trihydroxy-11-octadecenoic acid, were most abundant and accumulated in CBL. Eight lipids, including 1-α-linolenoyl-glycerol-2, 3-di-O-glucoside, 2-α-linolenoyl-glycerol-1-O-glucoside, 1-α-linolenoyl-glycerol-3-O-glucoside, 2-linoleoylglycerol, choline alfoscerate, linoleic acid, 1-oleoyl-Sn-glycerol, and 4-Oxo-9Z, 11Z, 13E, 15E-octadecatetraenoic acid, were most enriched in JSQ. Six lipids, including PE (oxo-11:0/16:0), 2-linoleoylglycerol-1, 3-di-O-glucoside, 2-linoleoylglycerol-1-O-glucoside, 1-linoleoylglycerol-3-O-glucoside, 1-(9Z-octadecenoyl)-2-(9-oxo-nonanoyl)-sn-glycero-3-phosphocholine and 2-aminoethylphosphonic acid, were most abundant in SDQ. 2-α-Linolenoyl-glycerol and 1-α-linolenoyl-glycerol were most abundant in NX. Ethyl 9-hydroxy-10, 12-octadecadienoic acid was most abundant in LL (Supplementary Table S12).

## Discussion

Radish is an important economic crop worldwide. Previous studies on radish metabolites mainly focused on specific classes of metabolites, such as glucosinolates, anthocyanin and flavonoids (8, 11, 31–33). However, the differences in metabolic profiles of radish cultivars have not been fully understood until now. In this study, we performed a UPLC-MS/MS-based targeted metabolomic analysis to understand the differences in taste between eight representative landraces, with various shape, color, and uses. A total of 938 metabolites were identified, and more than 300 metabolites were significantly and differentially accumulated in TXH and the other landraces. Therefore, this study provides novel insights for understanding the taste differences among the taproots of different radish landraces.

Each landrace has a specific metabolic profile, making it unique in taste and different in use. TXH with red skin and red flesh, is widely used in food coloring and pigment extraction industries. In our study, 19 anthocyanins (such as pelargonidin- and cyanidin-based derivatives) were significantly abundant in TXH, which was consistent with a previous study (11). Furthermore, 21 carbohydrates and 14 organic acids were significantly enriched in TXH, which contribute to the desirable taste with slight sweet and sour flavors (Brix 7.00%, pH 5.49) (Supplementary Tables S6–12).

MSH and LL, with red taproot skin, are widely used in kimchi and cooking. In our study, 9 lipids, 4 organic acids, 3 amino acid, 3 polyphenols, 3 carbohydrates, and 3 amino acids were significantly abundant in MSH, making it distinctive from the other landraces. Twelve organic acids, 5 GS, 4 amino acids, 2 sugars, 1 lipid, and biotin were most abundant in LL, which could explain the slightly pungent taste (Brix 4.80%, pH 6.09) (Supplementary Tables S6–12).

CHB and CBL, both with white skin and white flesh, are widely used in cooking and stewing. In our study, 12 kinds of organic acids, 11 carbohydrates, 9 lipids, 7 amino acids, and 5 GS were most abundant in CHB, which contribute to its sweet and sour taste (Brix 5.20%, pH 5.91). Eight lipids and three carbohydrates were most abundant in CBL, resulting in a mild taste (Brix 4.30%, pH 5.87) (Supplementary Tables S6–12).

SDQ and JSQ with green skin and green flesh are widely used as fruits and vegetables. In our study, 9 GS, 6 lipids, 3 carbohydrates, 3 amino acids, and 2 organic acids were significantly abundant and accumulated in SDQ, which results in a strong pungent and crisp taste (Brix 6.60%, pH 5.94). Thirty-four amino acid, 8 lipids, 6 organic acids, and 2 vitamins were most abundant in JSQ, contributing to its sweet and crisp flavors (Brix 7.00%, pH 5.93) (Supplementary Tables S6–12). NX, with green-white skin and flesh, is widely used in cooking and processing. In our study, 7 amino acids, 4 vitamins, 2 lipids, and 2 organic acids were most abundant in NX, which impart a light sweet crisp taste (Brix 6.10%, pH 5.74) (Supplementary Tables S6–12).

## Conclusions

In this study, UPLC-MS/MS-based metabolic analysis was performed successfully to systematically compare taste differences between the taproot of TXH and the other landraces. This work provides new insights into the differences in compositions and abundances of metabolites in radish taproot. We proposed that the difference in composition and concentrations of glucosinolates, carbohydrates, organic acids, amino acids, polyphenols, vitamins, and lipids might be the underlying causes of the differences in taste among the taproot of different radish landraces.

## Data availability statement

The original contributions presented in the study are included in the article/Supplementary Material, further inquiries can be directed to the corresponding author.

## Author contributions

Conceptualization, writing–original draft, and writing–review and editing: JZ and SM. Vol data analysis: ZH. All authors contributed to the article and approved the submitted version.

## Funding

This work was supported by the National Key Research and Development Program of China (No. 2017YFD0101806), the Agricultural Talents Program of the Chinese Academy of Agricultural Sciences (No. CAASQNYC-KYYJ-38), the Natural Science Foundation of Hunan Province (No. 2020JJ5642), and the Central Public-interest Scientific Institution Basal Research Fund (No. 1610242021008).

## Conflict of interest

The authors declare that the research was conducted in the absence of any commercial or financial relationships that could be construed as a potential conflict of interest.

## Publisher's note

All claims expressed in this article are solely those of the authors and do not necessarily represent those of their affiliated organizations, or those of the publisher, the editors and the reviewers. Any product that may be evaluated in this article, or claim that may be made by its manufacturer, is not guaranteed or endorsed by the publisher.
