# Supplementary material for: Identification and analysis of major flavor compounds in radish taproots by widely targeted metabolomics
Source: Front Nutr. 2022 Jul 18;9:889407. doi: 10.3389/fnut.2022.889407 (PMC9340154; doi:10.3389/fnut.2022.889407)
Supplement: Supplementary file 1 [file Data_Sheet_1.pdf]

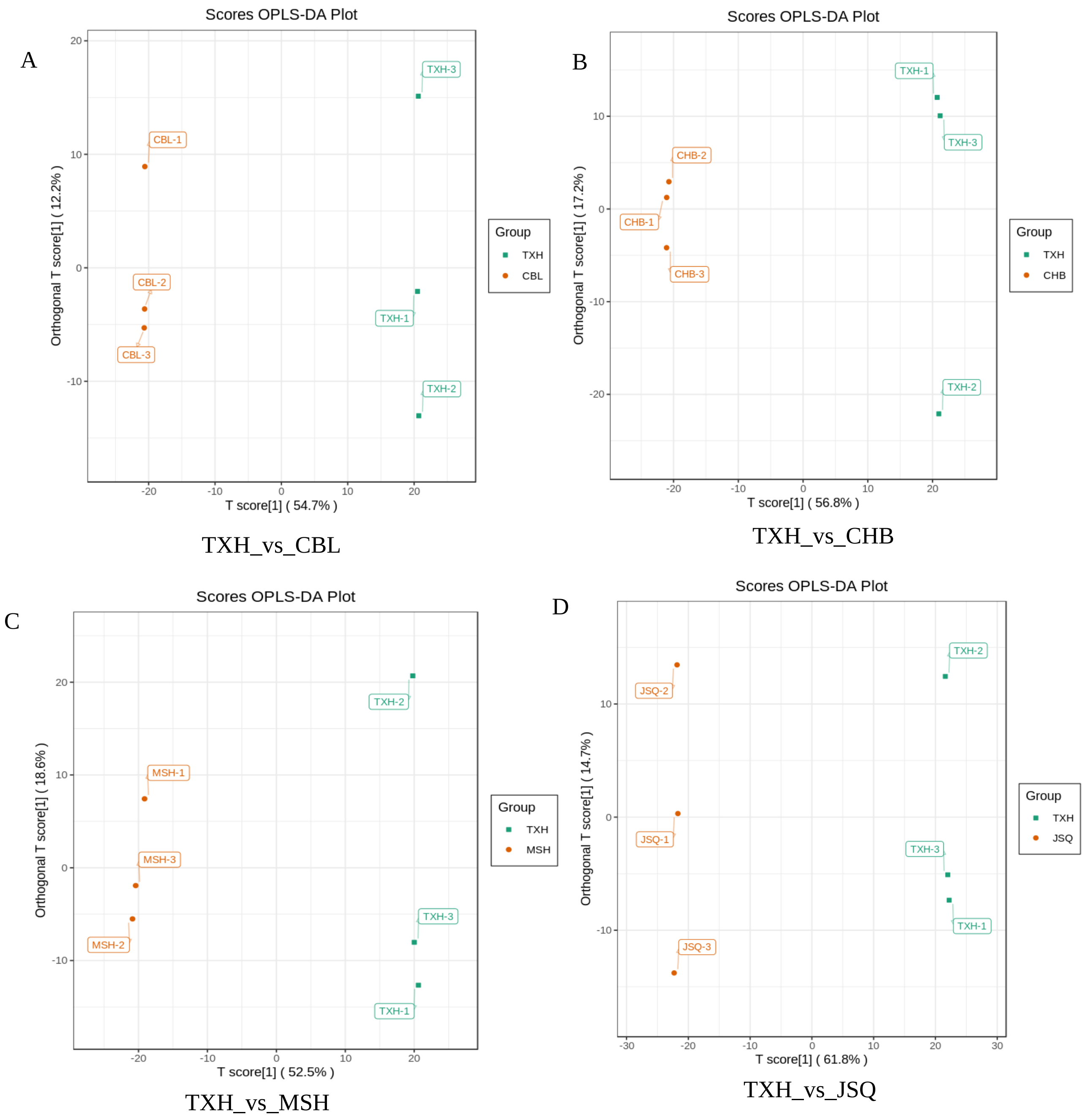
A
Scores OPLS-DA Plot
Orthogonal T score[1] (12.2%)
T score[1] (54.7%)
TXH-3
TXH-1
TXH-2
CBL-1
CBL-2
CBL-3
Group
TXH
CBL
TXH_vs_CBL
B
Scores OPLS-DA Plot
Orthogonal T score[1] (17.2%)
T score[1] (56.8%)
TXH-1
TXH-3
TXH-2
CHB-2
CHB-1
CHB-3
Group
TXH
CHB
TXH_vs_CHB
C
Scores OPLS-DA Plot
Orthogonal T score[1] (18.6%)
T score[1] (52.5%)
TXH-2
TXH-3
TXH-1
MSH-1
MSH-3
MSH-2
Group
TXH
MSH
TXH_vs_MSH
D
Scores OPLS-DA Plot
Orthogonal T score[1] (14.7%)
T score[1] (61.8%)
TXH-2
TXH-3
TXH-1
JSQ-2
JSQ-1
JSQ-3
Group
TXH
JSQ
TXH_vs_JSQ

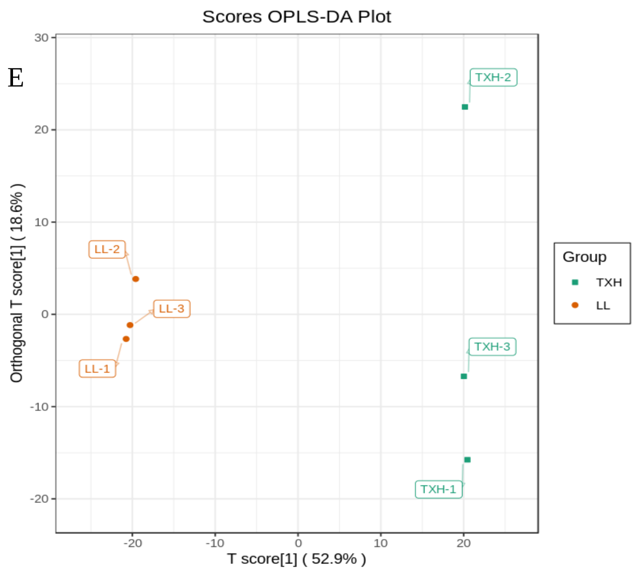

TXH_vs_LL

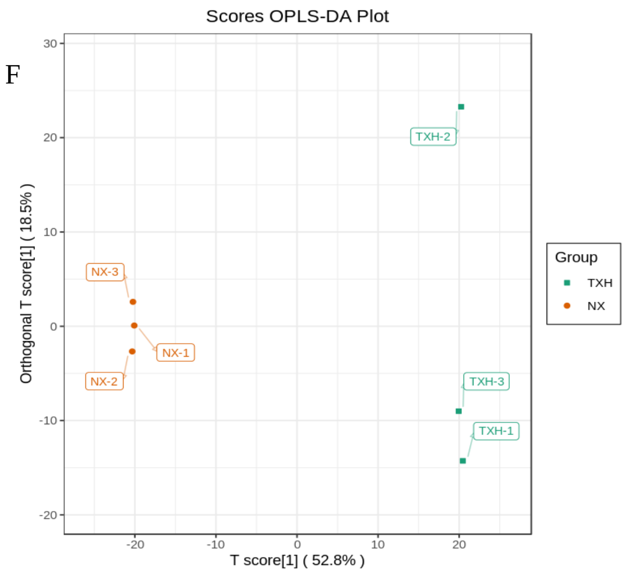

TXH_vs_NX

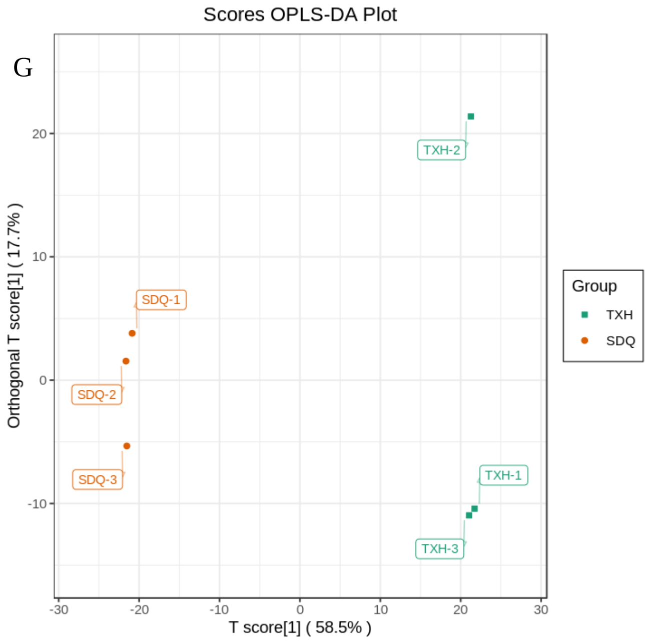

TXH_vs_SDQ

Figure S1 OPLS-DA model plots of CBL (A), CHB (B), MSH (C), JSQ (D), LL (E), NX (F) and SDQ (G) compared to TXH.

A

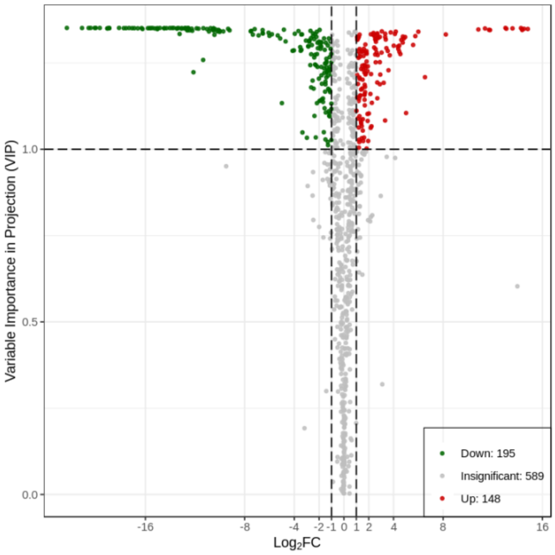

TXH_vs_CBL

B

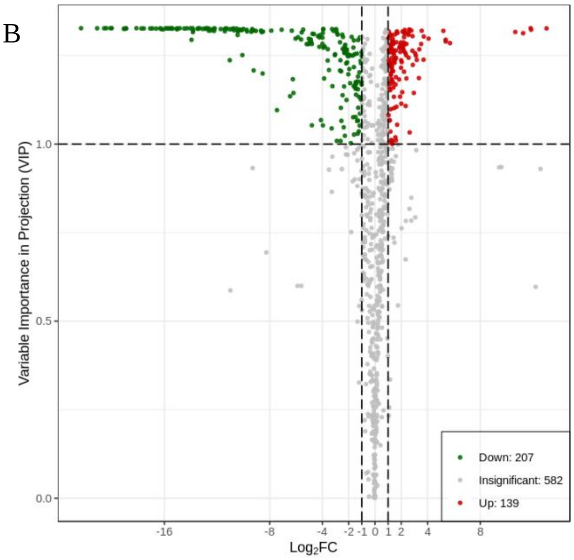

TXH_vs_CHB

C

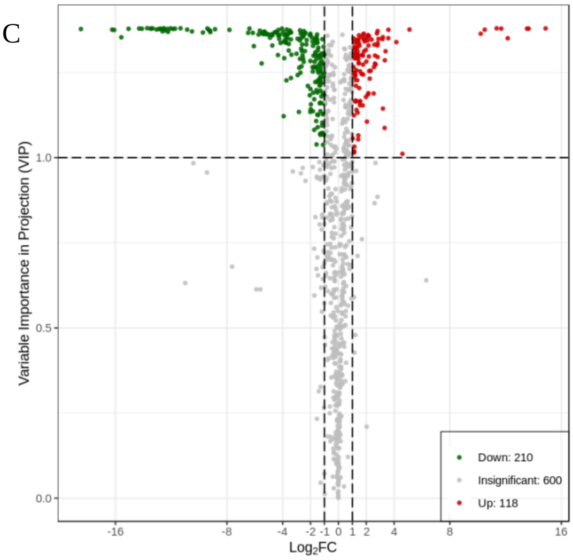

TXH_vs_MSH

D

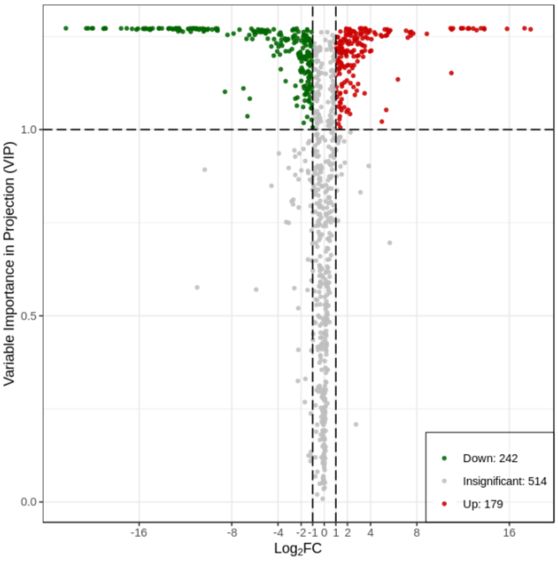

TXH_vs_JSQ

E

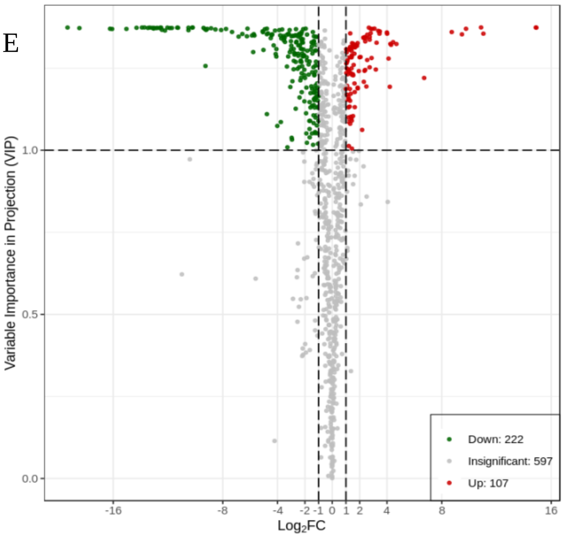

TXH_vs_LL

F

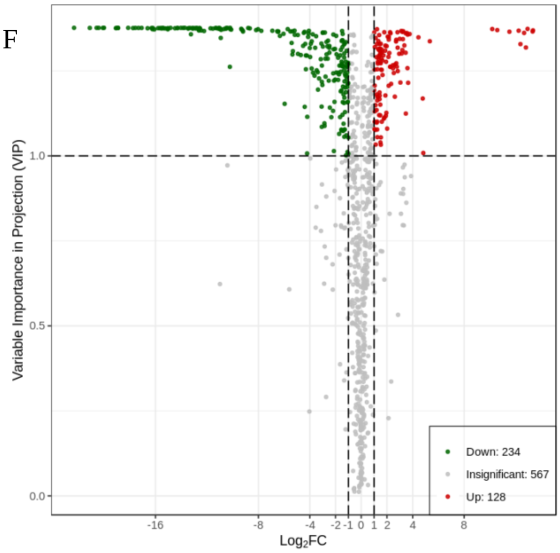

TXH_vs_NX

G

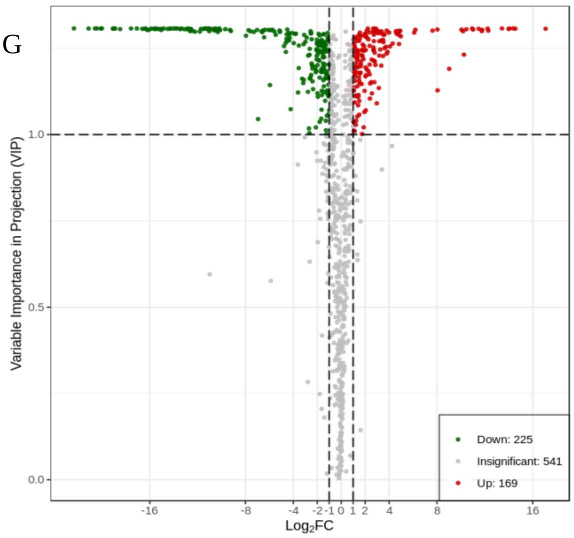

TXH_vs_SDQ

Figure S2 Volcano plots displaying differential metabolites between CBL (A), CHB (B), MSH (C), JSQ (D), LL (E), NX (F), SDQ (G), and TXH. Green spots illustrate lower differential metabolites; red spots show higher differential metabolites; gray spots display insignificant differential metabolites.

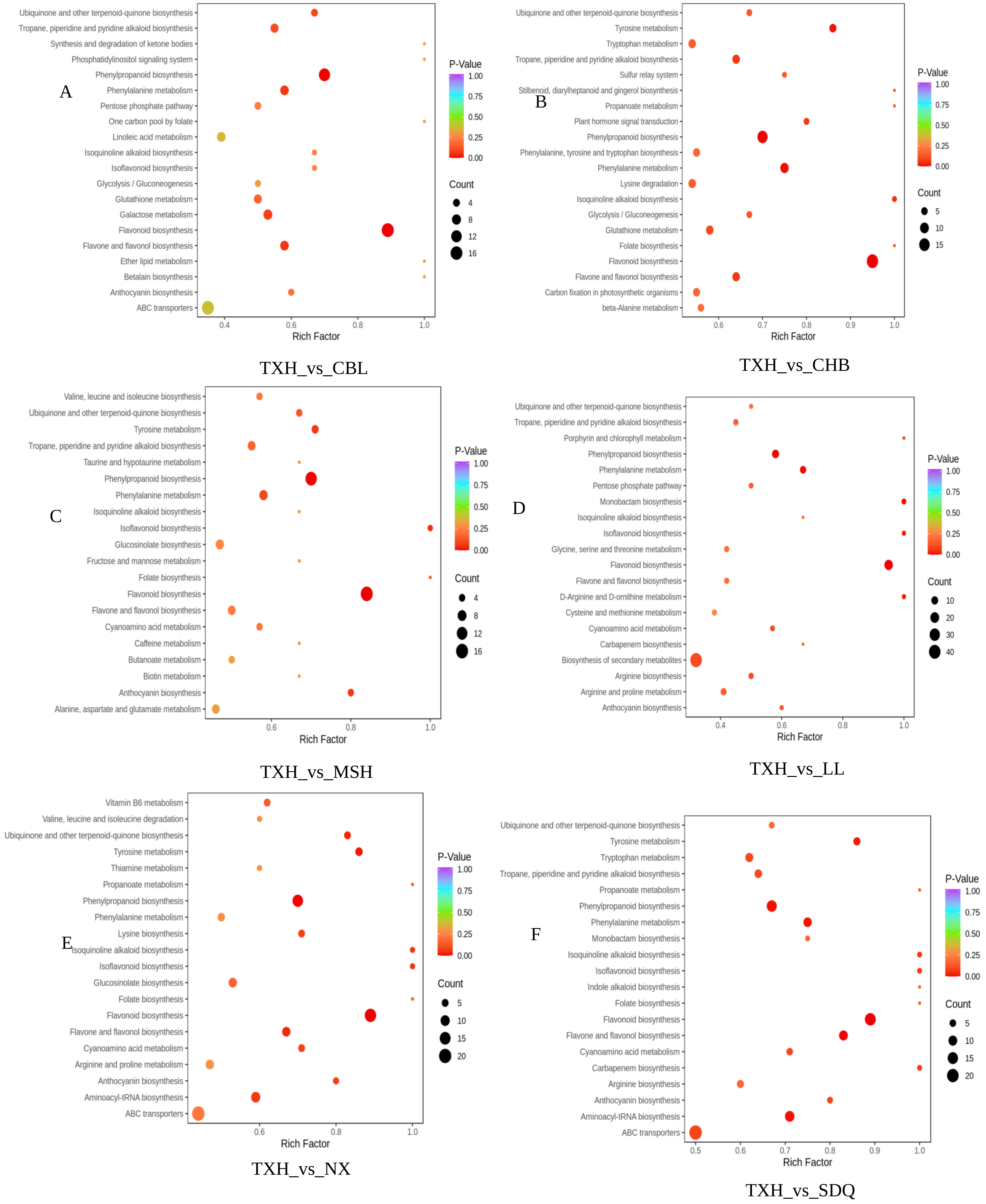
A
Ubiquinone and other terpenoid-quinone biosynthesis
Tropane, piperidine and pyridine alkaloid biosynthesis
Synthesis and degradation of ketone bodies
Phosphatidylinositol signaling system
Phenylpropanoid biosynthesis
Phenylalanine metabolism
Pentose phosphate pathway
One carbon pool by folate
Linoleic acid metabolism
Isoquinoline alkaloid biosynthesis
Isoflavonoid biosynthesis
Glycolysis / Gluconeogenesis
Glutathione metabolism
Galactose metabolism
Flavonoid biosynthesis
Flavone and flavonol biosynthesis
Ether lipid metabolism
Betalain biosynthesis
Anthocyanin biosynthesis
ABC transporters
0.4 0.6 0.8 1.0
Rich Factor
P-Value 1.00 0.75 0.50 0.25 0.00
Count 4 8 12 16
TXH_vs_CBL
B
Ubiquinone and other terpenoid-quinone biosynthesis
Tyrosine metabolism
Tryptophan metabolism
Tropane, piperidine and pyridine alkaloid biosynthesis
Sulfur relay system
Stilbenoid, diarylheptanoid and gingerol biosynthesis
Propanoate metabolism
Plant hormone signal transduction
Phenylpropanoid biosynthesis
Phenylalanine, tyrosine and tryptophan biosynthesis
Phenylalanine metabolism
Lysine degradation
Isoquinoline alkaloid biosynthesis
Glycolysis / Gluconeogenesis
Glutathione metabolism
Folate biosynthesis
Flavonoid biosynthesis
Flavone and flavonol biosynthesis
Carbon fixation in photosynthetic organisms
beta-Alanine metabolism
0.6 0.7 0.8 0.9 1.0
Rich Factor
P-Value 1.00 0.75 0.50 0.25 0.00
Count 5 10 15
TXH_vs_CHB
C
Valine, leucine and isoleucine biosynthesis
Ubiquinone and other terpenoid-quinone biosynthesis
Tyrosine metabolism
Tropane, piperidine and pyridine alkaloid biosynthesis
Taurine and hypotaurine metabolism
Phenylpropanoid biosynthesis
Phenylalanine metabolism
Isoquinoline alkaloid biosynthesis
Isoflavonoid biosynthesis
Glucosinolate biosynthesis
Fructose and mannose metabolism
Folate biosynthesis
Flavonoid biosynthesis
Flavone and flavonol biosynthesis
Cyanoamino acid metabolism
Caffeine metabolism
Butanoate metabolism
Biotin metabolism
Anthocyanin biosynthesis
Alanine, aspartate and glutamate metabolism
0.6 0.8 1.0
Rich Factor
P-Value 1.00 0.75 0.50 0.25 0.00
Count 4 8 12 16
TXH_vs_MSH
D
Ubiquinone and other terpenoid-quinone biosynthesis
Tropane, piperidine and pyridine alkaloid biosynthesis
Porphyrin and chlorophyll metabolism
Phenylpropanoid biosynthesis
Phenylalanine metabolism
Pentose phosphate pathway
Monobactam biosynthesis
Isoquinoline alkaloid biosynthesis
Isoflavonoid biosynthesis
Glycine, serine and threonine metabolism
Flavonoid biosynthesis
Flavone and flavonol biosynthesis
D-Arginine and D-ornithine metabolism
Cysteine and methionine metabolism
Cyanoamino acid metabolism
Carbapenem biosynthesis
Biosynthesis of secondary metabolites
Arginine biosynthesis
Arginine and proline metabolism
Anthocyanin biosynthesis
0.4 0.6 0.8 1.0
Rich Factor
P-Value 1.00 0.75 0.50 0.25 0.00
Count 10 20 30 40
TXH_vs_LL
E
Vitamin B6 metabolism
Valine, leucine and isoleucine degradation
Ubiquinone and other terpenoid-quinone biosynthesis
Tyrosine metabolism
Thiamine metabolism
Propanoate metabolism
Phenylpropanoid biosynthesis
Phenylalanine metabolism
Lysine biosynthesis
Isoquinoline alkaloid biosynthesis
Isoflavonoid biosynthesis
Glucosinolate biosynthesis
Folate biosynthesis
Flavonoid biosynthesis
Flavone and flavonol biosynthesis
Cyanoamino acid metabolism
Arginine and proline metabolism
Anthocyanin biosynthesis
Aminoacyl-tRNA biosynthesis
ABC transporters
0.6 0.8 1.0
Rich Factor
P-Value 1.00 0.75 0.50 0.25 0.00
Count 5 10 15 20
TXH_vs_NX
F
Ubiquinone and other terpenoid-quinone biosynthesis
Tyrosine metabolism
Tryptophan metabolism
Tropane, piperidine and pyridine alkaloid biosynthesis
Propanoate metabolism
Phenylpropanoid biosynthesis
Phenylalanine metabolism
Monobactam biosynthesis
Isoquinoline alkaloid biosynthesis
Isoflavonoid biosynthesis
Indole alkaloid biosynthesis
Folate biosynthesis
Flavonoid biosynthesis
Flavone and flavonol biosynthesis
Cyanoamino acid metabolism
Carbapenem biosynthesis
Arginine biosynthesis
Anthocyanin biosynthesis
Aminoacyl-tRNA biosynthesis
ABC transporters
0.5 0.6 0.7 0.8 0.9 1.0
Rich Factor
P-Value 1.00 0.75 0.50 0.25 0.00
Count 5 10 15 20
TXH_vs_SDQ

G

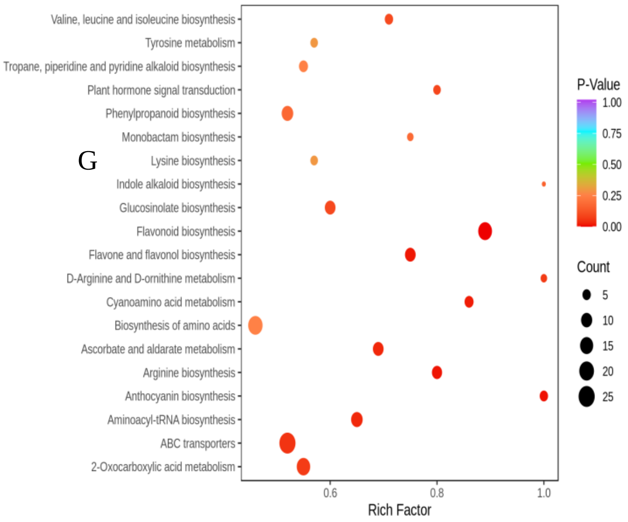

TXH_vs_JSQ

Figure S3 The enrichment analysis of identified metabolites involved in KEGG pathway. (A) TXH vs CBL; (B) TXH vs CBL; (C) TXH vs MSH; (D) TXH vs LL; (E) TXH vs NX; (F) TXH vs SDQ; (G) TXH vs JSQ.
